# The association between renal accumulation of pancreatic amyloid-forming amylin and renal hypoxia

**DOI:** 10.3389/fendo.2023.1104662

**Published:** 2023-02-16

**Authors:** Nirmal Verma, Florin Despa

**Affiliations:** Department of Pharmacology and Nutritional Sciences, University of Kentucky, Lexington, KY, United States

**Keywords:** kidney disease, mitochondria, reactive oxygen species, hypoxia, amylin

## Abstract

Chronic kidney disease (CKD) is increasing worldwide and is associated with diabetic states (obesity, prediabetes and type-2 diabetes mellitus). The kidney is intrinsically susceptible to low oxygen (hypoxia) and renal hypoxia plays a vital role in the progression of CKD. Recent studies suggest an association between CKD and renal deposition of amyloid-forming amylin secreted from the pancreas. Renal accumulation of amyloid-forming amylin is associated with hypertension, mitochondrial dysfunction, increased production of reactive oxygen species (ROS) and activation of hypoxia signaling in the kidney. In this review we will discuss potential associations between renal amylin amyloid accumulation, hypertension, and mechanism of hypoxia-induced kidney dysfunction, including activation of hypoxia-inducible factors (HIFs) and mitochondrial dysfunction.

## 1 Introduction

The prevalence of kidney disease is increasing worldwide (1). According to the Centers for Disease Control and Prevention’s (CDC) Chronic Kidney Disease in the United States, 2021 report nearly 786,000 people in the United States are living with end-stage renal disease, with 71% on dialysis and 29% with a kidney transplant (2).

Renal hypoxia plays an important role in the progression of kidney disease through mechanisms that involve activation of hypoxia-inducible factor (HIF), a master regulator of cellular adaptation to hypoxia (1, 3, 4). Renal hypoxia is closely associated with the development of renal inflammation and fibrosis, and is common in diabetic nephropathy, anemia, cardiovascular diseases, and sarcopenia (5, 6).

The kidney is intrinsically susceptible to hypoxia. It uses only 10% of oxygen delivered by the renal artery (7). Kidney diseases are characterized by renal fibrosis and gradual decline in the glomerular filtration rate (GFR) or both. Hypoxia is a condition in which organs or cells lack a sufficient amount of oxygen supply (8). The formation of hypoxic status is determined by various factors, low oxygen supply, high energy demand, and cellular resistance to hypoxia. In the kidney, proximal tubular cells are the most sensitive to hypoxic injury and the extent of tubular injury determines the prognosis of kidney disease (9). In response to hypoxia, pericytes detach from the vessel wall and differentiate into activated myofibroblasts in interstitial space, leading to development of renal fibrosis or renal injury (10). In addition, hypoxia also induces endothelial cells activation, followed by leukocyte stasis and blocking blood supply to peritubular capillaries leading to loss of capillaries and exacerbating hypoxia and loss of nephrons (11).

Mitochondria are essential organelles and play an important role in the physiology of all organs including kidneys. Mitochondria produce cellular energy in the form of ATP which is supplied to all cells to perform their normal function. During mitochondrial metabolism, reactive oxygen species (ROS) are produced. In normal conditions ROS function as secondary messengers, inducing redox-sensitive post-translational modifications (PTM) in proteins and activating or deactivating different cell signaling pathways. However, in pathological conditions such as kidney diseases, ROS overproduction causes oxidative stress (OS) and hypoxia, inducing mitochondrial dysfunction and altering its metabolism and dynamic. The latter processes are closely related to changes in the cell redox-sensitive signaling pathways, causing inflammation and apoptosis cell death (12). For its normal function kidney is required a huge amount of energy, which is supplied by mitochondria (13, 14). Therefore, any dysfunction affecting mitochondria will also have a crucial impact on renal cellular function.

Amylin is a 37 amino acid long pancreatic hormone and co-secreted with insulin from beta cells (15–18). Studies from our lab and from other labs showed amylin have novel function in renal function and path-physiology and also in other organs (19–24). A recent study from our lab showed that red blood cell (RBC)-capillary interaction is altered by prediabetic hypersecretion of amylin that could be a potential contributing factor to renal hypoxia in diabetic kidney injury (19). Following findings from other labs also showed a link between amylin and renal physiology: 1) presence of high affinity amylin binding sites in renal cortex (20), 2) *in vivo* injection of radiolabeled amylin showed presence of amylin binding site on proximal tubules of kidney (22), 3) administration of amylin peptide in human and rats, stimulated plasma renin many folds (20, 25–27), 4) Amylin is a potent stimulator of sodium and water reabsorption in kidney (22), 5) Amylin acts as mitogen, stimulating hyperplasia of epithelial cells of proximal tubules (22).

In this review we will discuss potential associations between renal amylin amyloid accumulation and mechanism of hypoxia-induced kidney dysfunction, including HIF activation and mitochondrial dysfunction.

## 2 Hypoxia inducible factors (HIFs) and hypoxia signaling

**Figure 1:**
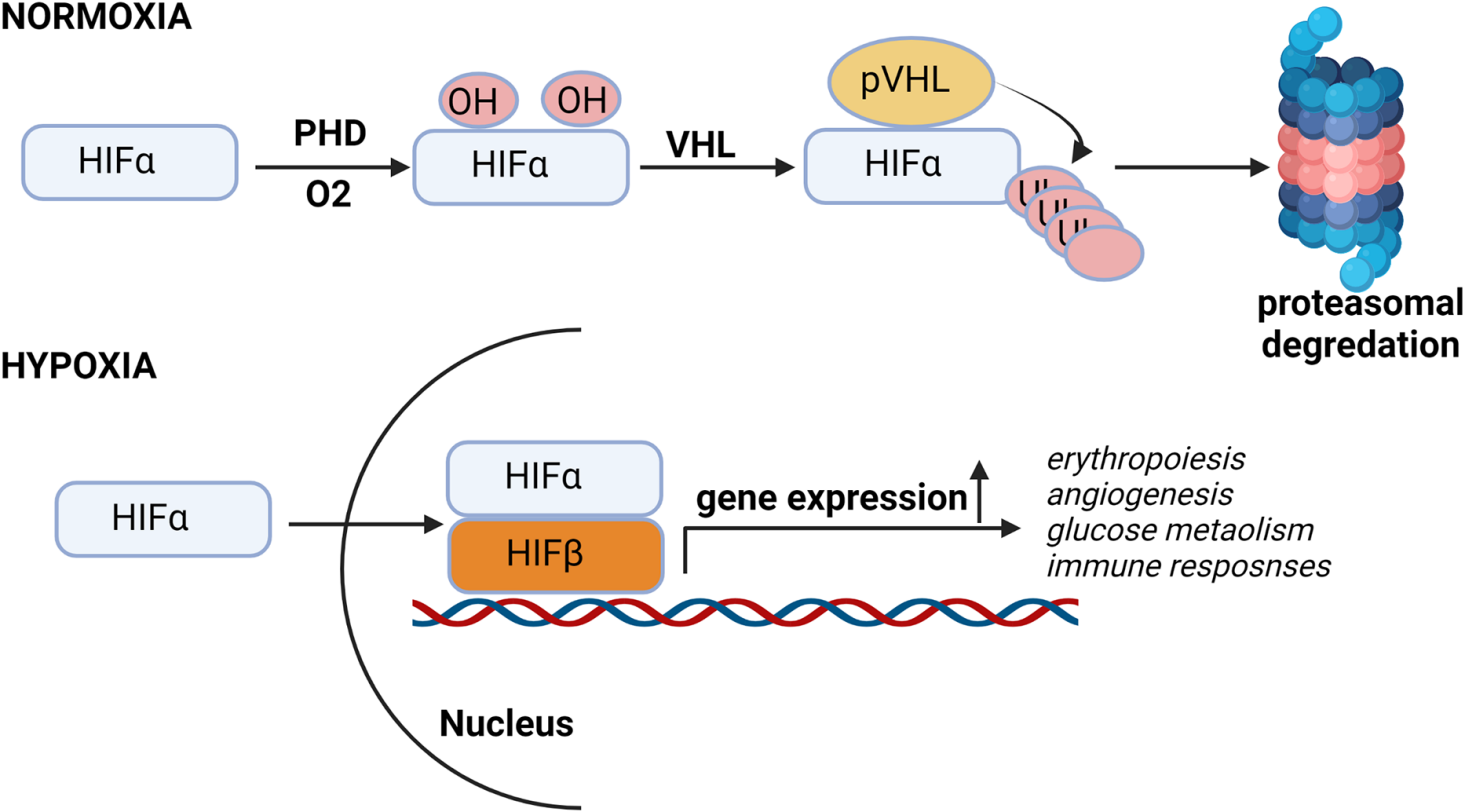
Hypoxia pathway under normal oxygen and hypoxic conditions. In presence of oxygen, HIF-α subunits are hydroxylated by oxygen-dependent prolyl-4-hydroxylases (PHDs) and then Von Hippel–Lindau protein (pVHL), an E3 ubiquitin ligase, binds to the hydroxylated HIF-α and, which leads to the proteasomal degradation of HIF protein. Under low oxygen conditions, HIF is stabilized and translocated into the nucleus, where it binds to its dimerization partner HIF1β and enhances the transcription of HIF target genes.

Kidney cells as well as other cells in body are adopted for low oxygen condition or hypoxia through stabilization of HIFs. HIFs are transcription factors responsible for the induction of genes (Erythropoiesis, angiogenesis, glucose metabolism, apoptosis and immune responses) essential for survival under hypoxia conditions (**Figure 1**).

HIF is heterodimer of constitutively expressing β subunit and an oxygen regulated α subunit. The α subunit are synthesized continuously irrespective of the oxygen status of the cells. Under normal oxygen concentration or normoxia, enzyme prolyl Hydroxylase domain (PHD) hydroxylates proline residues of HIFα. Proline hydroxylated HIFα is recognized by the Von Hippel Lindau (VHL) an E3 ubiquitin ligase complex, resulting in HIFα ubiquitination and subsequent proteasomal degradation (**Figure 1**) (28–30).

## 3 Mechanism of hypoxia-induced hypoxic-ischemic injury in kidney

**Figure 2:**
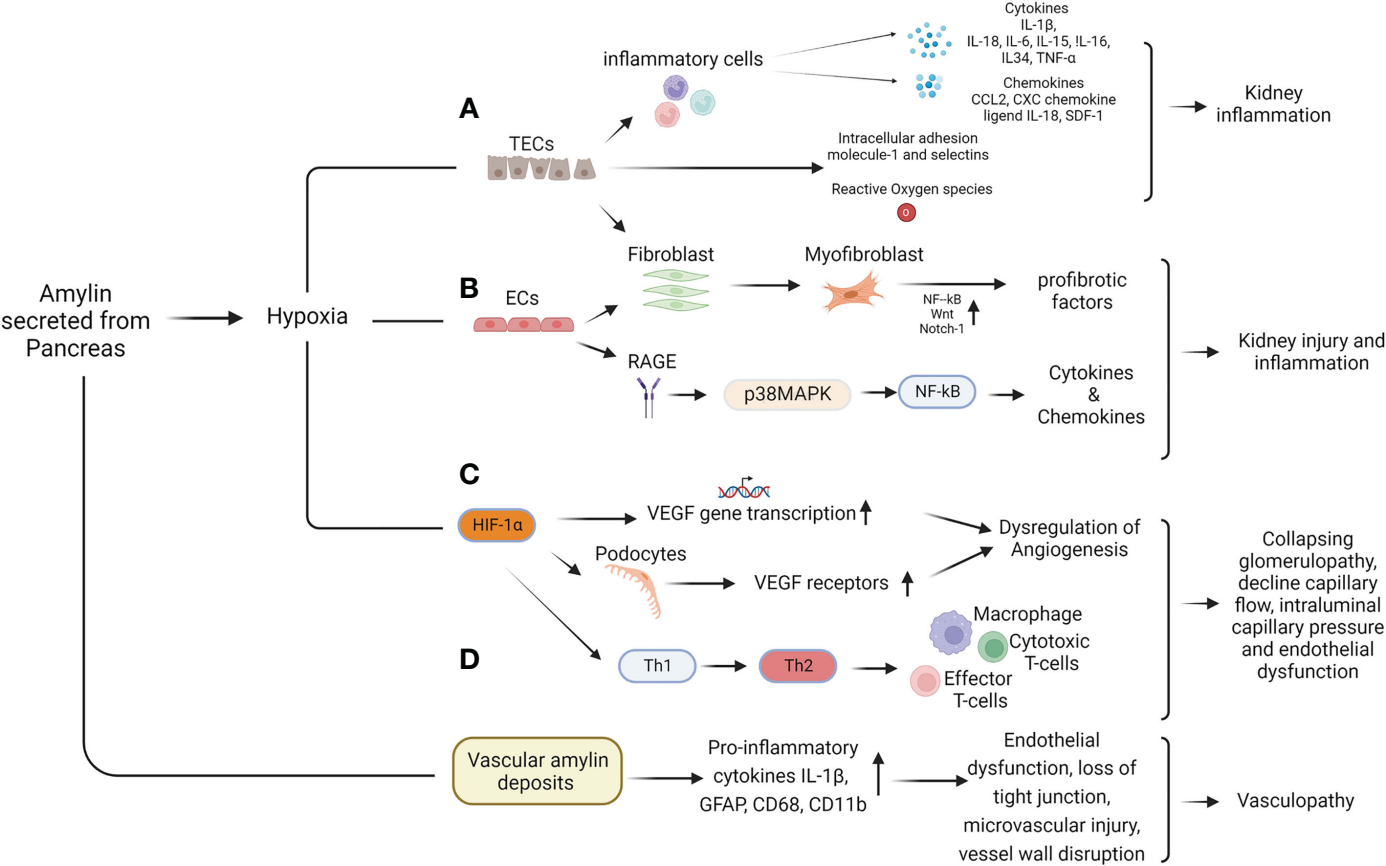
Mechanism of Hypoxia induced kidney pathologies. Figure shows hypoxia induce kidney damage involves multiple pathways, including RAGE, p38 MAPK, EMT, dysregulation of angiogenesis and inflammation. Under hypoxia condition, renal fibroblasts changed into myofibroblasts and causes increased ECM synthesis to induce renal fibrosis **(A)**. In low oxygen endothelial also trans-differentiates in to myofibroblasts and causes kidney fibrosis. PTE cells are sensitive to hypoxic environments, and NF-κB, Wnt and Notch-1 signaling can be activated to trigger inflammatory cytokines, chemokines, adhesion molecules and peritubular inflammation to promote fibrosis **(B)**. HIFs promote angiogenesis dysregulation by regulating the gene transcription, mRNA, and protein expression of VEGF and VEGF receptors resulted in renal damage **(C)**. Recruitment of proinflammatory cells and cytokines, phenotypic transition of T cells induced by HIF-1α, differentiation and proliferation of regulatory T cells and dendritic cells, etc. are promoters of myofibroblast activation that affect angiogenesis, resulting in collapsing glomerulopathy, decreased capillary flow, intraluminal capillary pressure, and endothelial dysfunction, which in turn aggravates hypoxia **(C)**. Beside Hypoxia, vascular amylin deposition also causes inflammation, endothelial dysfunction and microvascular injury resulted in amylin vasculopathy **(D)**.

The renal proximal tubules are packed with mitochondria and dependent on oxidative phosphorylation, and are vulnerable to various oxidative injury like hypoxia. In response to hypoxia, tubular epithelial cells (TECs) undergoes changes and start functioning like inflammatory or fibrogenic cells (31). Transformed TECs can facilitate the inflammatory response through production of various bioactive molecules such as pro-inflammatory cytokines (Interleukins, tumor necrosis factors, colony stimulating factors and growth factors), chemokines (monocyte chemoattractant protein-1/CCL2, CXC chemokine ligand 8/IL-8 and CXC chemokine ligand 12/SDF-1), adhesion molecules (intracellular adhesion molecue-1 and selectins), reactive oxygen species and C-reactive proteins which can lead to interstitial inflammation in kidney (**Figure 2A**).

Under hypoxia condition TECs could also undergo changes in structure and phenotypes that are accompanied by altered expression and production of profibrotic factors causing tubulointerstitial fibrosis (TIF). As the fibrosis increase tubular capillary network becomes spares and leading to a decreased blood supply and declined renal function (31, 32) (**Figure 2A**).

Renal endothelial cells (ECs) are another main target of hypoxia. Hypoxia in kidney contribute to renal disease progression by activating the receptor for advance glycation end products (RAGE) and stimulating p38 MAPK and NFkB downstream signaling in ECs. (33) (**Figure 2B**). Under hypoxia injury, endothelial cells could also differentiate in to myofibroblasts (EndoMT), which further increase the production of extracellular matrix (ECM) and conversely aggravate hypoxia and hypoxia induced injury in kidney (**Figure 2B**).

Hypoxia also plays a critical role in epithelial-mesenchymal transition (EMT) in proximal tubular cells and activates NFkB signaling to trigger peritubular inflammation through stimulation of Wnt and Notch-1 signaling to promote kidney fibrosis (34–36) (**Figure 2B**).

Under hypoxia condition renal interstitial fibroblasts could also proliferate and differentiate in to myofibroblasts and promote renal scarring by accelerating extracellular matrix synthesis (**Figure 2B**) (34–36).

In chronic kidney disease hypoxia contributes to the development of peritubular capillary rarefaction and dysregulation of angiogenesis (37). Hypoxia induced dysregulation of angiogenesis in ECs is regulated by nitric oxide synthases, vascular endothelial growth factor (VEGF), and angiopoietins (38) and affecting the proliferation and migration of endothelial cells (**Figure 2C**). In addition, hypoxia induced HIF regulates angiogenesis related genes by increase activation of VEGF and internal ribosomal entry. Excessive activation of VEGF in podocytes under hypoxia condition causes collapsing glomerulopathy which resulting in decreased capillary flow and intraluminal capillary pressure. VEGF receptors (VEGFR) are predominantly expressed in endothelial cells in glomerular and peritubular capillaries and over expression of VEGFR in hypoxia also promotes endothelial dysfunction (39–42) (**Figure 2C**).

Under hypoxia condition, HIF-1α level is increased in T cells and induces phenotypic transition from type 1 helper T cells (Th1) to type 2 T cells (Th2) to amplify the immune response of macrophages and cytotoxic T cells. (43). Beside this increased HIF-1α in hypoxia condition also negatively regulate the adaptive immune system to protect tissues by activating the differentiation and proliferation of regulatory T cells and inhibit effector T cells (44) (**Figure 2D**). Hypoxia also inhibit the differentiation of dendritic cells to enhance the link between hypoxia and immune response in kidney.

## 4 Diabetes-associated hyperamylinemia and hypoxic-ischemic injury in kidney

**Figure 3:**
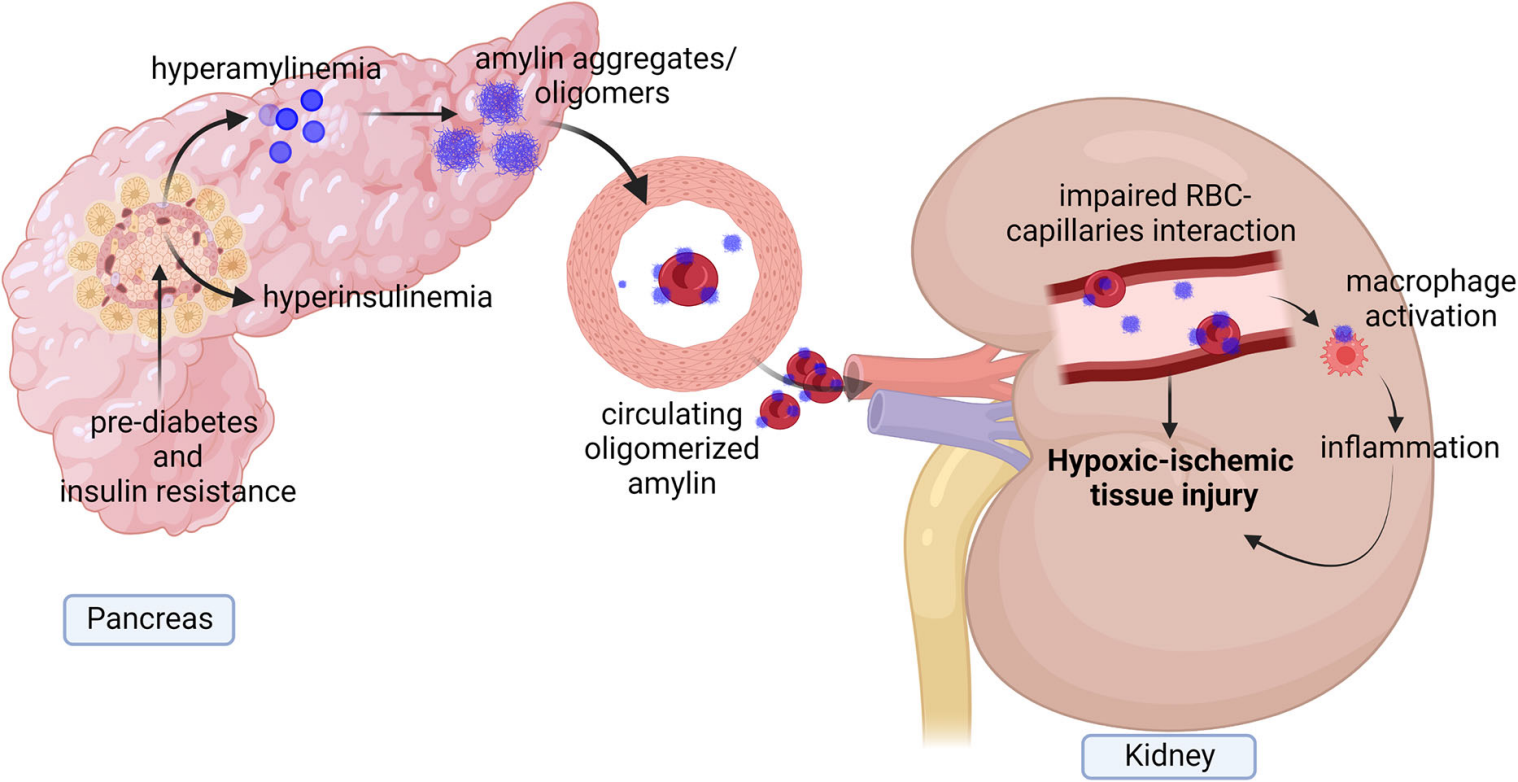
Diagram showing diabetes associate hyperamylinemia increases hypoxia signaling and inflammation in kidney. Prediabetes associated hyperamylinemia promotes aggregation of human amylin in pancreas. Human amylin aggregates also reach to circulation where they deposited in microvasculature and on Red blood cells (RBCs) leading to reduced RBCs capillaries interaction. Accumulation of human amylin aggregates in renal tissues and microvasculature promote elevation of EPO level, hypoxia markers and inflammation causing hypoxic injury of renal tissues.

Amylin from humans and a few other species, including cats, dogs, and monkeys, but not rodents, have an increased propensity to aggregate, forming amyloid (i.e., amylin dyshomeostasis) (18, 45–47). Hypersecretion of human amylin is known to activate HIF-1α, a marker of hypoxia signaling (48–50). Accumulating evidence demonstrates the presence of amylin amyloid deposition in heart, brain, and kidneys of patients with type 2 diabetes (51–57). Hypersecretion of human amylin is associated with amylin oligomers deposition in the microvasculature and red blood cells (RBCs) leading to impaired RBC capillaries interaction, increased plasma erythropoietin level and increased hypoxia markers (HIF-1α, HIF-2α, arginase-1, arginase-2 and arginase activity) causing the hypoxic-ischemic injury of renal tissues (19). Amylin deposition in kidney microvasculature also colocalized with macrophages activation which indicates that amylin dyshomeostasis injuries capillaries and associated with inflammatory responses exacerbating ischemic vascular injury in the kidney (**Figure 3**) (19). This indicates diabetes associated hypersecretion of amylin promotes deposition of amylin oligomers in kidney tissues which can increase hypoxia signaling pathway and inflammation leading to kidney injury and disease (**Figure 3**).

## 5 Renal hypoxia is associated with amylin-induced hypertension

Systemic hypertension is caused by the chronic induction of multiple vasoconstrictions including the renin-angiotensin-aldosterone system (58, 59). As the blood vessels constrict, blood flow to kidney tissues is reduced, consequently reducing the oxygen supply to the kidneys (60–63). Besides this hypertension causes the kidney to consume more oxygen compared to normal for the transport of the same amount of sodium (61, 63). Thus, hypertension induces lower renal oxygenation or renal hypoxia through a combination of a reduced supply of oxygen caused by vasoconstriction and increased oxygen demand. It was also reported that hypertension predisposes the kidney to kidney failure by inducing renal hypoxia (64, 65), and detrimental effects of hypoxia are exacerbated by hypertension, rendering renal tissue to produce elevated levels of reactive oxygen species (66). Increased ROS in kidney tissues elevates angiotensin receptors that transduce signals to activate the pro-oxidant enzyme NADPH-oxidase (NOX) (66, 67). ROS produced by hypertension acts in the same way that generated during diabetic kidney diseases drives renal hypoxia injury. Studies with drugs against hypertension in CKD patients also showed renal hypoxia is associated with hypertension. (63, 67–71).

Previous studies have found a link between increased levels of renal amylin and amylin binding sites with increased renal hypertension and thus diabetes-associated hypersecretion of amylin could be involved in hypertension-induced renal hypoxia. High-affinity binding sites for amylin have been reported in kidneys and are involved in the genesis of hypertension (72). Rat models of hypertension, injected with labeled amylin peptide showed an increase in the density of amylin binding sites in the kidney even before the actual increase in systolic blood pressure compared to normal rats (72). These rats showed a further increase in amylin binding sites with the development of systolic blood pressure. Histological examination of kidneys from these rats showed the presence of elevated amylin binding sites in proximal tubules. Further studies in the rat models of renal ablation and hypertension showed systolic blood pressure is correlated with the density of amylin binding in the cortex. Thus, changes in amylin levels and amylin binding sites with renal hypertension showed a possible role of amylin in the development of renal hypertension. The same group of researchers also showed inhibition of angiotensin-converting enzyme reduces the density of amylin binding sites in kidney tissues besides reducing systolic blood pressure which also show a link between amylin and renal hypertension (73). Overall, we can postulate that increased levels of renal hypoxia in diabetes could be caused by amylin-induced hypertension.

## 6 Mitochondrial reactive oxygen species (mtROS)

**Figure 4:**
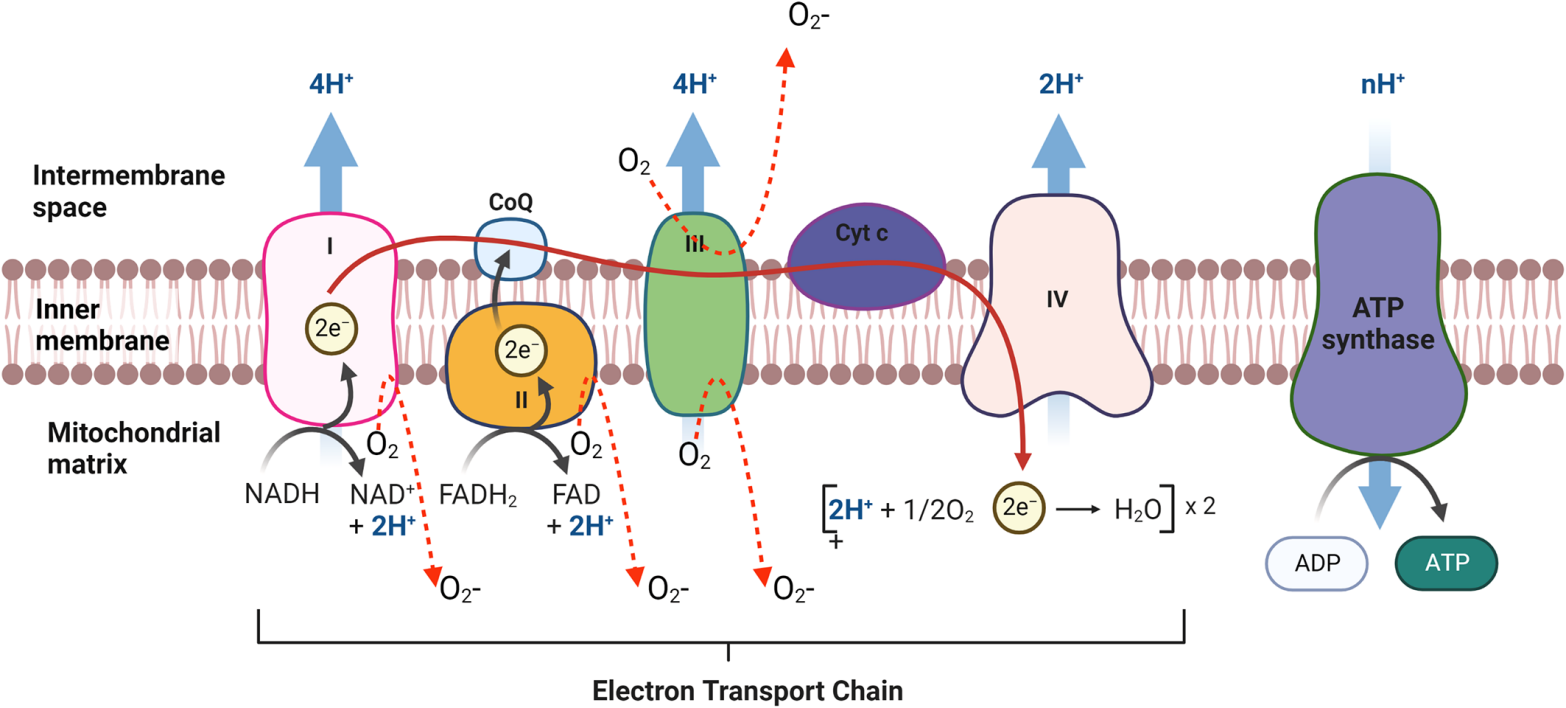
mtROS production in Electron transport chain (ETC) in mitochondria. The ETC is located in the mitochondrial inner membrane (IM). Complex I and II supply electrons to coenzyme Q (CoQ; ubiquinone). Sequentially, electrons are transferred from CoQ to Complex III, cytochrome c (Cyt c) and Complex IV. Oxidative stress is generated during electron transfer.

Mitochondria are the major contributor to ROS production (~90% of cellular ROS) (74, 75). mtROS produce at the electron transport chain (ETC) during the oxidative phosphorylation of molecular oxygen (O_2_) to reduced H_2_O (76–78). During their transport, electron leak and interact with molecular oxygen to form superoxide (O_2_^-.^) at complex I and the Q cycle of complex III, which are major sources of superoxide and H_2_O_2_ in mitochondria (**Figure 4**) (78–81). In the first step of ETC, complex I transfer two electrons from nicotine adenine dinucleotide (NADH) to ubiquinone (Q) from low to high potential and reduces ubiquinone to ubiquinol (QH_2_) (82, 83). During this process, mtROS can be generated in the matrix by complex I (83, 84). Complex III is the major site of ROS generation at the ETC. Complex III has an inner (Qi) and outer (Qo) pools of ubiquinone oriented towards the matrix and at the intermembrane space respectively (85, 86). Ubisemiquinone at complex III is the primary direct electron donor capable of reducing O2 to superoxide. Ubisemiquinone carries a single electron which can move freely in complex III and directly leak the single electron to O_2_ resulting in ROS generation. Although complex I and complex III are the primary production sites in mitochondria, complex II may produce ROS to a lesser extent. The FAD site of complex II can produce O_2_^-^ toward the matrix but the production rate of ROS at complex II is very low compared to complex I and complex III (**Figure 4**) (83, 86).

Other sites in mitochondria except for the ETC, may produce mtROS. The mitochondrial glycerol 3-phosphate dehydrogenase (mGPDH), which oxidized the glycerol 3-phosphate and reduces Q to QH_2_ resulting in feeding electrons into ETC is capable of generating ROS in mitochondria (87–89). Another site where electrons could escape and form ROS in mitochondria is the electron transferring flavoprotein-ubiquinone oxidoreductase (ETF-QOR) (90). Other sources of mtROS include pyruvate dehydrogenase 2-oxoglutarate dehydrogenase (Odh), dihydroorotate dehydrogenase, and p66shc/cytochrome c (87, 88).

Cellular components other than mitochondria are also capable of producing ROS in kidney. NADPH oxidases (NOX) are accepted as a major source of ROS generating in kidney (91, 92). This family is composed of seven members from NOX1 (colon), NOX2 (phagocytes), NOX3 (inner ear), NOX4, NOX5 (lymphoid tissues), DUXO1 and DUXO2 (thyroid and bronchus). NOX4 is expressed predominantly in kidney and is associated with various renal complications (91, 93, 94).

## 7 Mitochondrial ROS, HIF stabilization and hypoxic-ischemic injury in kidney

Kidney disease or kidney injury is a condition when the glomerular filtration rate (GFR) is decreased to less than 60ml/min per 1.73m^2^ or shows the presence of markers for kidney damage or both (95). The most common causes of kidney disease are diabetes and hypertension (95). The kidney needs a large amount of energy to maintain the body’s fluid composition by filtering and reabsorbing materials. Reabsorption requires a huge amount of energy in the form of ATP supplied by mitochondria (13) and thus mitochondria dysfunction will have a crucial impact on kidney function. Overproduction of mtROS in mitochondria (mtROS) is linked to mitochondrial dysfunction and oxidative stress and hypoxia, which is an early event of hypoxic-kidney injury. Mitochondrial dysfunction during kidney disease preceded podocyte fusion and proteinuria and result in epithelial cells to mesenchymal transition of renal tubular cells. (96, 97). Mitochondrial dysfunction not only precedes kidney injury but also contributes to a large increase in oxidative stress and hypoxia and to the development and progression of hypoxic-kidney injury due to loss of mitochondrial membrane potential and a drop in ATP production (98). Mitochondrial dysfunction has been linked to increasing in mtROS. Hydroxyl radicals can damage macromolecules in mitochondria such as mtDNA. Unrepaired damage of mtDNA can lead to defects in complex III, which results in an increased production of ROS and oxidative or hypoxic-kidney damage (99). Increased in oxidative damage can result in releasing intermembrane proteins to the cytosol such as cytochrome c and amplifying oxidative stress in the kidney, which gives rise to a viscous cycle of excessive mtROS production and mitochondrial dysfunction (99–101).

The first indication suggesting mitochondria act like oxygen sensors came when ρ0 Hep3B cells which are deficient in mitochondrial DNA, and thus no electron transport, are incapable of HIF-1α DNA binding activity and thus do not produce Erythropoietin (EPO) in response to low oxygen (102). Another finding where antioxidant treatment abolished the stabilization of HIF-1α under hypoxia also suggested that mitochondrial ROS is responsible for hypoxia signaling or HIFs stabilization under low oxygen conditions (102). Further, treatment of cells with H_2_O_2_ or inducing H_2_O_2_ production in cells or mutations that lead to H_2_O_2_ accumulation in cells is sufficient to increase HIF-1α even in normoxia (103). Embryonic cells lacking cytochrome c fails to stabilize HIF-1α under low oxygen condition also showed mitochondrial ROS is responsible for hypoxia-induced HIF-1α stabilization (104). The use of mitochondrial inhibitors showed ROS generation at complex III but not at complex I or II is critical for hypoxia-induced HIF-1α stabilization (105). Studies, where normal cells were fused with mitochondrial deficient cells, showed that it is not the ability of cells to convert oxygen or conduct oxidative phosphorylation but the ability of cells to produce ROS at mitochondrial complex II that is critical for the HIF-1α stabilization to downward hypoxia signaling.

**Figure 5:**
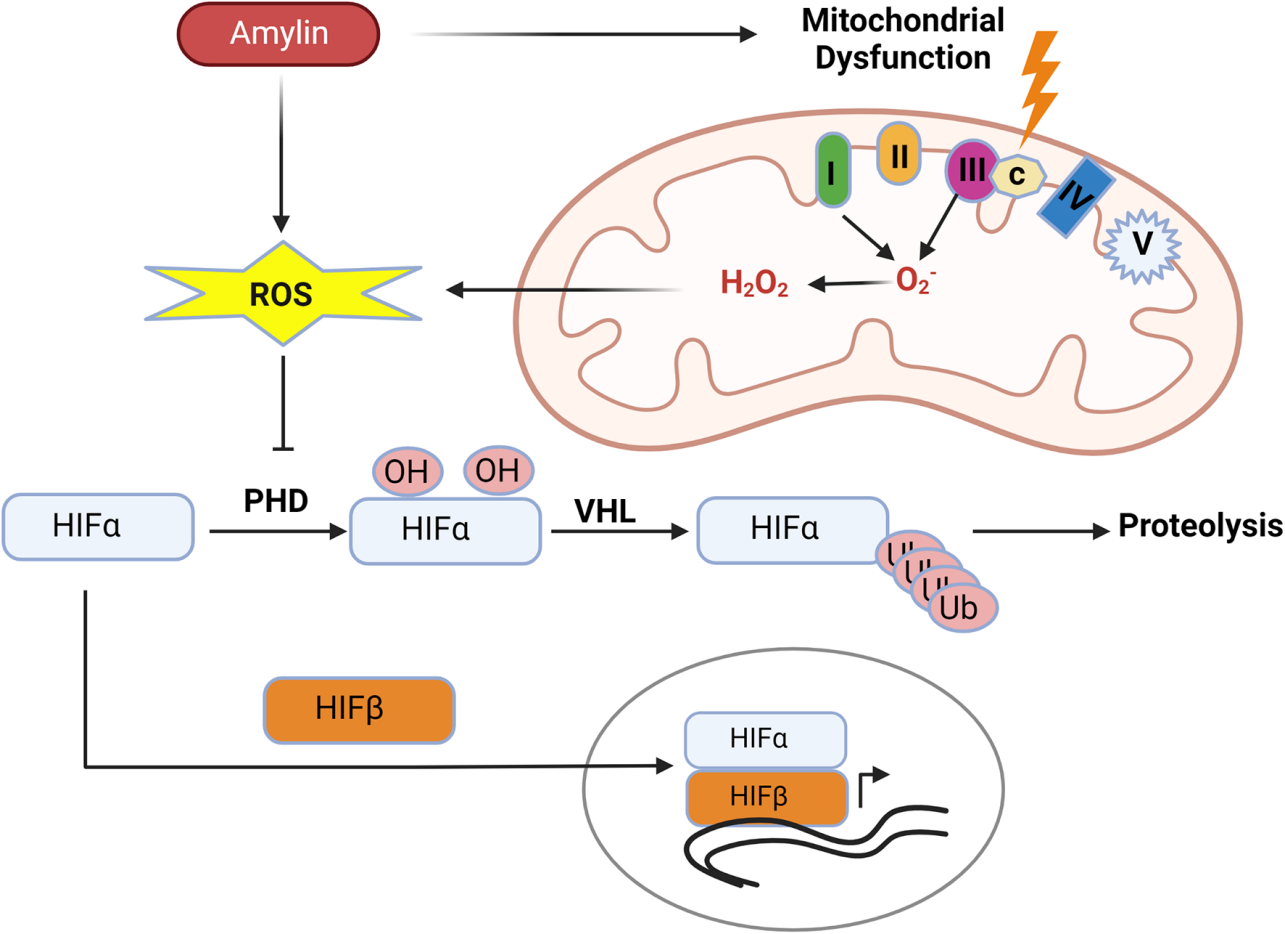
Human amylin induced mitochondrial dysfunction promotes hypoxia. Human amylin induced mitochondrial dysfunction increases production of mtROS which inhibits the activity of PHDs allowing for stabilization of HIF-1α subunits and HIF mediated transcription.

Thus, an increase in mtROS production resulting from mitochondrial dysfunction in kidney disease can cause stabilization of HIF-1α and hypoxia signaling in the kidney (**Figure 5**).

## 8 Diabetes associated hyperamylinemia, mitochondria dysfunction and mitochondrial ROS

Research has shown that human IAPP or amylin oligomers are cytotoxic and associated with endoplasmic reticulum stress, mitochondrial dysfunction, and mitochondrial ROS (106, 107). *In vitro* study with INS1F cells showed exogenous human amylin induces mitochondrial dysfunction and cell apoptosis (107). Mitochondrial peptidase pitrilysin regulates human amylin in beta cells’ mitochondria, and the intra-mitochondrial pool of amylin causes beta-cell apoptosis and mitochondrial dysfunction (108). Thus, diabetes-associated hyperamylinemia could promote hypoxic-renal injury by creating mitochondrial dysfunction and ROS. (**Figure 5**).

## 9 Perspectives: Pancreatic amyloid-forming amylin as a therapeutic target in CKD

Hypoxia is a critical mediator of the progression of kidney pathologies. Therefore, elucidating the response of kidney to hypoxia and factors that promote hypoxia is of a great significance to understand pathophysiology of kidney disease. Hypersecretion of amyloid-forming amylin is common in persons with prediabetes leading to deposition of aggregated amylin oligomers in the microvasculature of kidney and on RBCs. Amyloid-forming amylin impairs oxygen sensing at RBCs-capillary interface promoting activation of hypoxia signaling pathway in kidney (19), which may induce mitochondrial dysfunction through increasing mtROS generation. Future research is needed to identify inhibitors of amylin-induced hypoxia signaling in renal tissues as a potential therapeutic strategy to counteract the impact of diabetes on kidney function.

## Author contributions

All authors listed have made a substantial, direct, and intellectual contribution to the work, and approved it for publication.
